# Supplementary material for: The Discovery of Actinospene, a New Polyene Macrolide with Broad Activity against Plant Fungal Pathogens and Pathogenic Yeasts
Source: Molecules. 2021 Nov 20;26(22):7020. doi: 10.3390/molecules26227020 (PMC8621364; doi:10.3390/molecules26227020)
Supplement: Supplementary file 1 [file molecules-26-07020-s001.zip › molecules-1468646-supplementary.pdf]

Supplementary Material

The discovery of actinospene, a new polyene macrolide with broad activity against plant fungal pathogens and pathogenic yeasts

Ying Tang [1], Cuiyang Zhang [1], Tianqi Cui [2], Ping Lei [1], Zhaohui Guo [1,*], Hailong Wang [2,*] and Qingshu Liu [1,*]

1 Hunan Institute of Microbiology, Changsha, 410009, Hunan, People's Republic of China

2 State Key Laboratory of Microbial Technology, Institute of Microbial Technology, Helmholtz International Lab for Anti-infectives, Shandong University–Helmholtz Institute of Biotechnology, Shandong University, Qingdao, Shandong, People's Republic of China

* Correspondence: 1214837569@qq.com (Z.G.); wanghailong@sdu.edu.cn (H.W.); volcanoya@126.com (Q.L.)

Table S1. Gene annotation for the cryptic Actn BGC based on antiSMASH 6.0 prediction.

ORF	nt（bp）	Proposed function (domain organization)	Accession number
ActnS2	7474	TI PKS, KS-AT-DH-KR-PP-KS-AT	WP_035287518.1
ActnS0	8148	TI PKS, KS-AT-PP-KS-AT-DH-KR-PP	WP_035287521.1
ActnK	1431	Glycosyltransferase family 1 protein	WP_052021789.1
ActnC	1059	DegT/DnrJ/EryC1/StrS family aminotransferase	WP_035287523.1
ActnG	1179	Cytochrome P450	WP_035287525.1
ActnF	192	Ferredoxin	WP_035287527.1
ActnS4	6012	TI PKS, KS-AT-DH-KR-PP-TE	WP_035287529.1
ActnS1	5385	TI PKS, KS-AT-DH-KR-PP	WP_052021790.1
ActnS3	27906	TI PKS, KS-AT-DH-KR-PP-KS-AT -KR-PP-KS-AT-KR-PP-KS-AT-KR-PP-KS-AT- KR-PP-KS-AT- KR-PP	WP_035287531.1
ActnA	1839	ABC transporter ATP-binding protein	WP_035287533.1
ActnB	1737	ABC transporter ATP-binding protein	WP_084176275.1
ActnD3	1176	Cytochrome P450	WP_152552277.1
ActnD2	1182	Cytochrome P450	WP_035287537.1
ActnU	1740	Acyl-CoA ligase	WP_035287549.1
ActnV	2202	Acyl-CoA dehydrogenase	WP_035287553.1
ActnR1	693	TetR/AcrR family transcriptional regulator	WP_035287558.1
ActnD1	1206	Cytochrome P450	WP_035287568.1
ActnR2	603	TetR/AcrR family transcriptional regulator	WP_052021798.1
ActnR3	1174	Sensor histidine kinase	WP_152552284.1
ActnJ	1032	GDP-mannose 4,6-dehydratase	WP_035288993.1

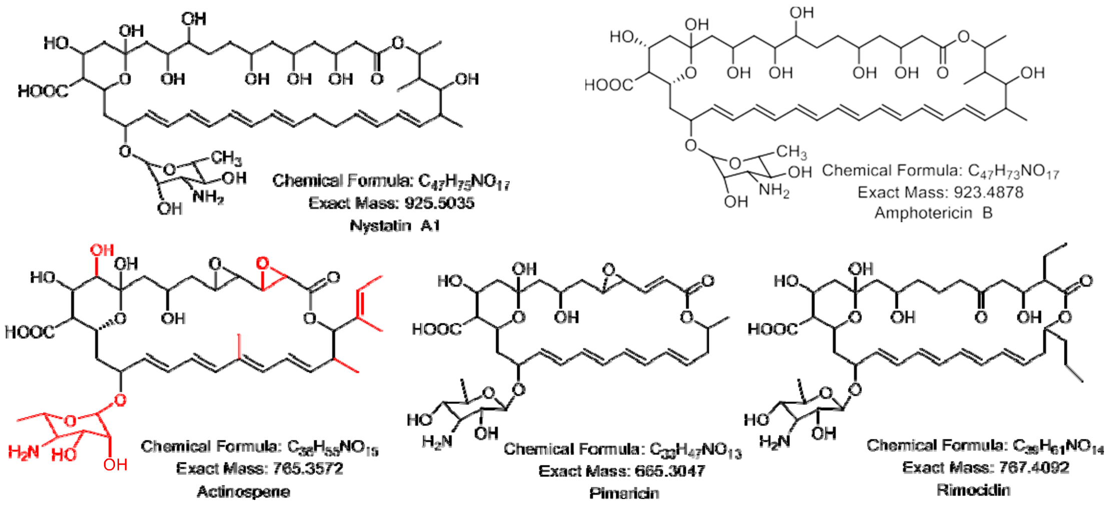

Figure S1. The planar structures of actinospene and previously reported polyene antifungal antibiotics. Differences between actinospene and pimaricin are indicated in red.

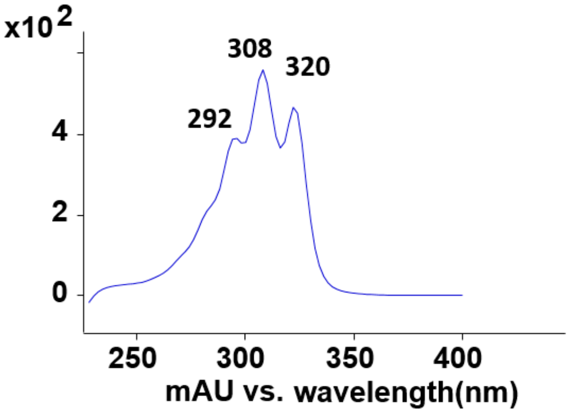

Figure S2. UV/Vis spectrum of actinospene (**1**).

UV/Vis spectrum of the main HPLC peak at t_R 5.27 min showing absorption maxima at 292, 308, and 320 nm.

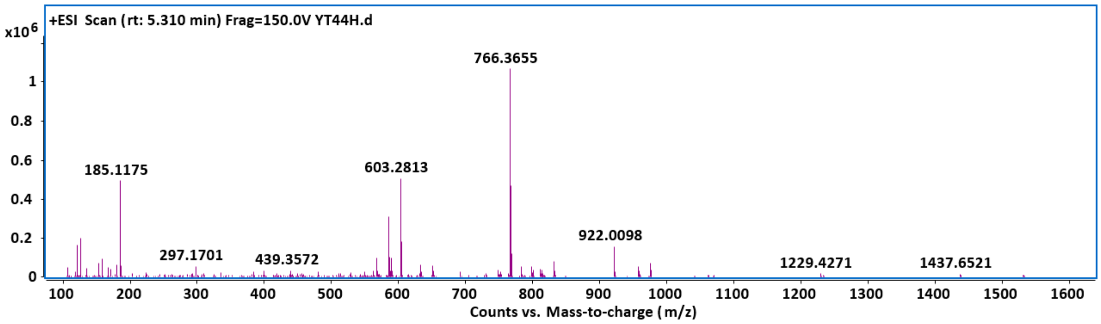

Figure S3. High resolution mass spectrum of actinospene (**1**).

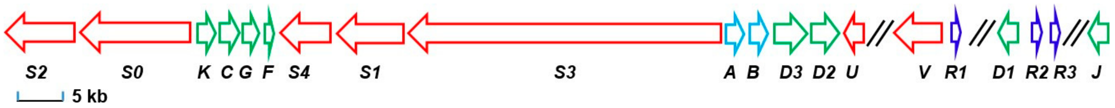

Figure S4. Schematic representation for the cryptic Actn BGC.

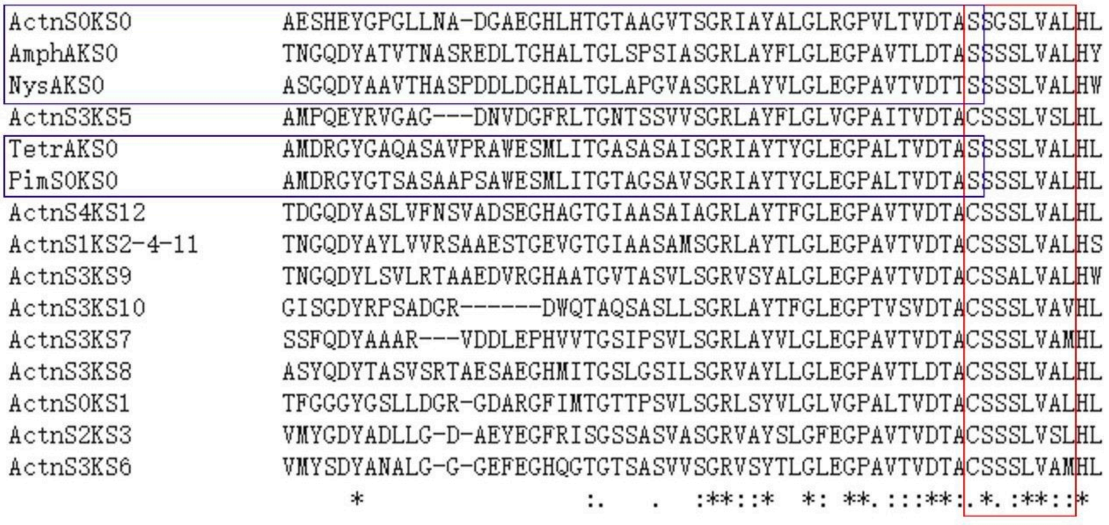

Figure S5. Partial amino acid sequence comparison for the KS domains.

The KS domains of the loading module of amphotericin, pimaricin, tetramycin, nystatin PKS all contain a serine instead of a cysteine in the active site as indicated by the cross region of the red and blue boxes. Abbreviations: Nys: Nystatin; Amph: Amphotericin; Pim: pimaricin; Tetr: tetramysin.

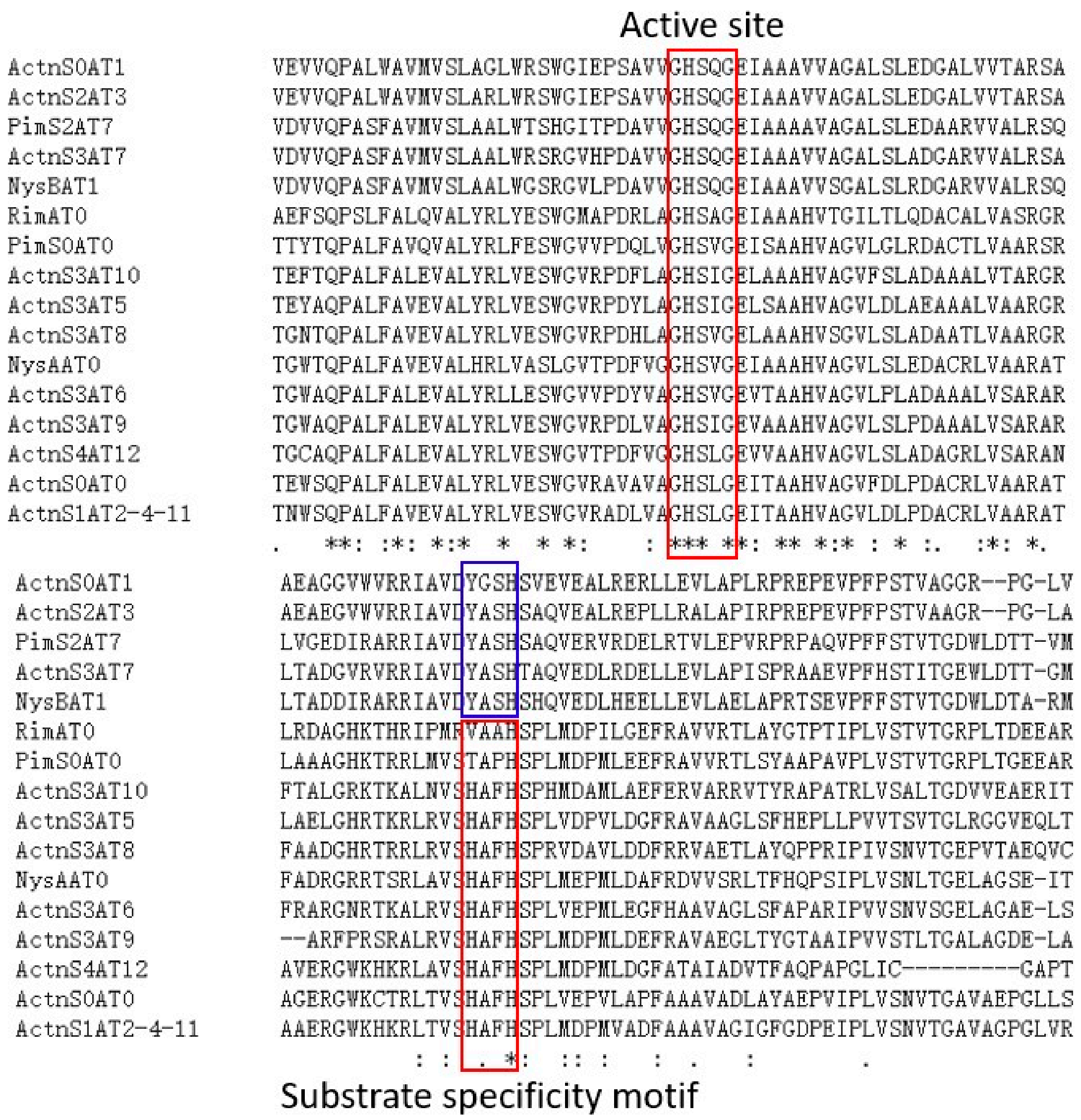

Figure S6. Partial sequence comparison for the AT domains.

Analysis of AT domains show that all of them possess a GHSXG motif at the active site. Malonyl-CoA specificity is determined by HAFH motif while YASH motif selects for methylmalonyl CoA as substrate [1].

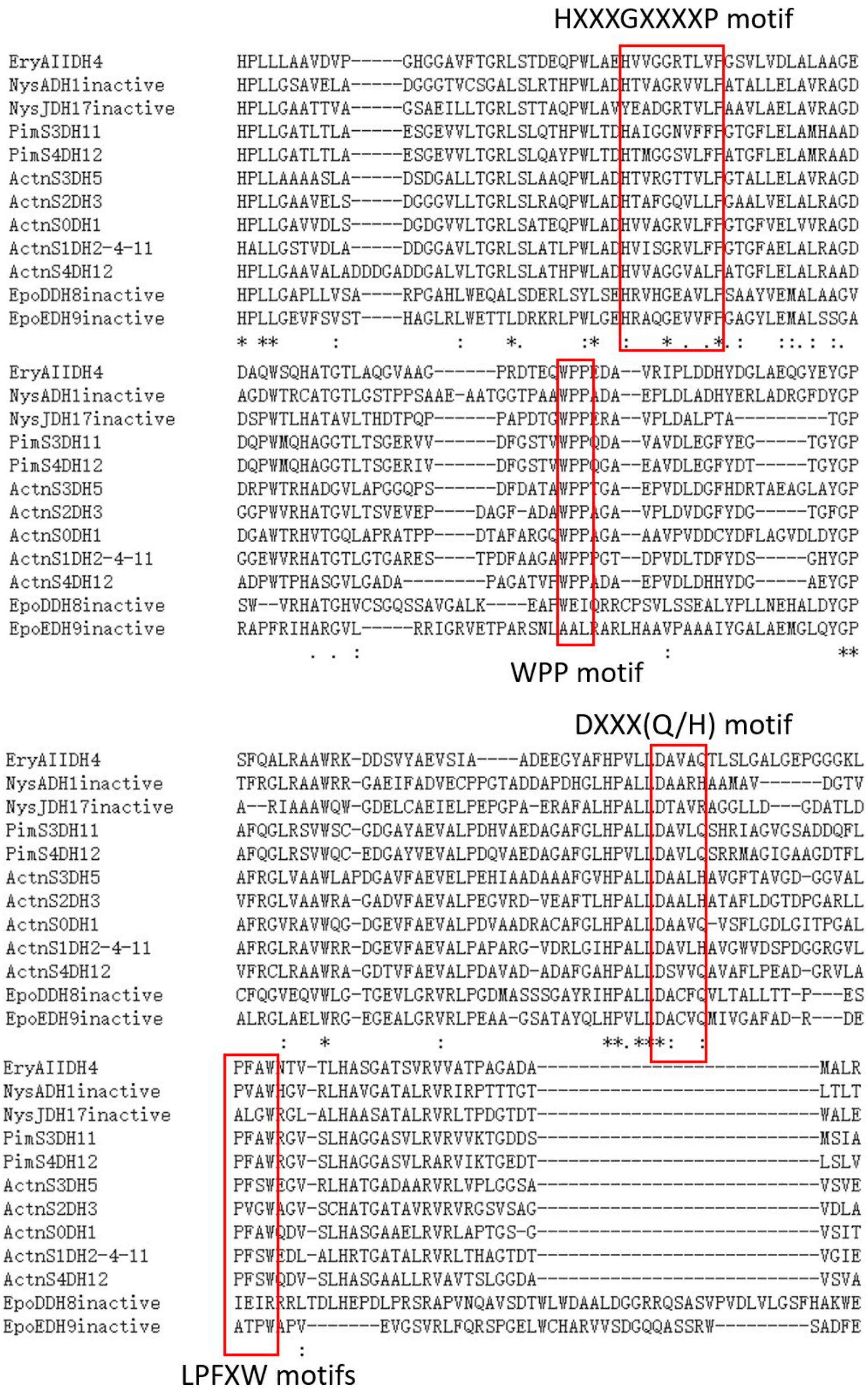

Figure S7. Partial sequence comparison for the DH domains.

All DH domains possess the crucial HXXXGXXXXP motif involved in the catalytic site[2]. Other conserved motif such as the "WPP" motif are point out by red boxes.

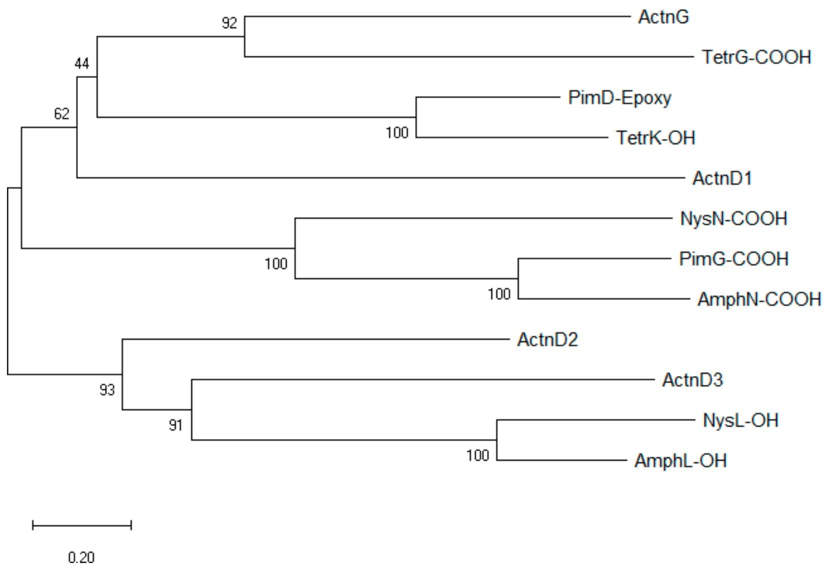

Figure S8. Phylogenetic analysis of the P450 by neighbor-Joining method.

Bootstrap values (expressed as percentage of 1000 replications) are shown at the branch points. Bar 0.2 substitutions per nucleotide position.

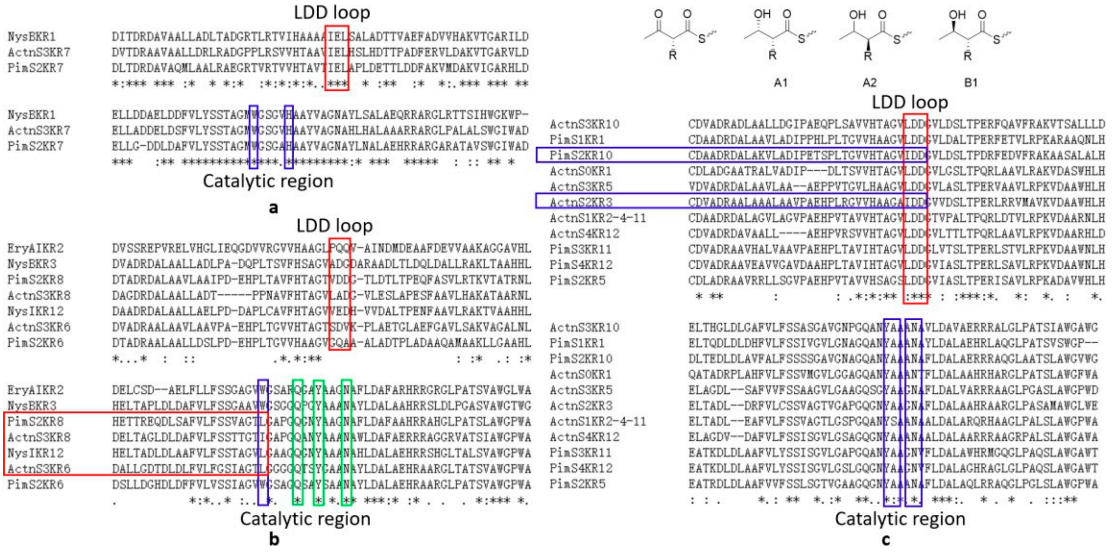

Figure S9. Partial sequence comparison for the KR domains.

Fingerprints featured for A1, A2, B1-type are highlighted by red and blue boxes [3].

Abbreviations: Ery: erythromycin.

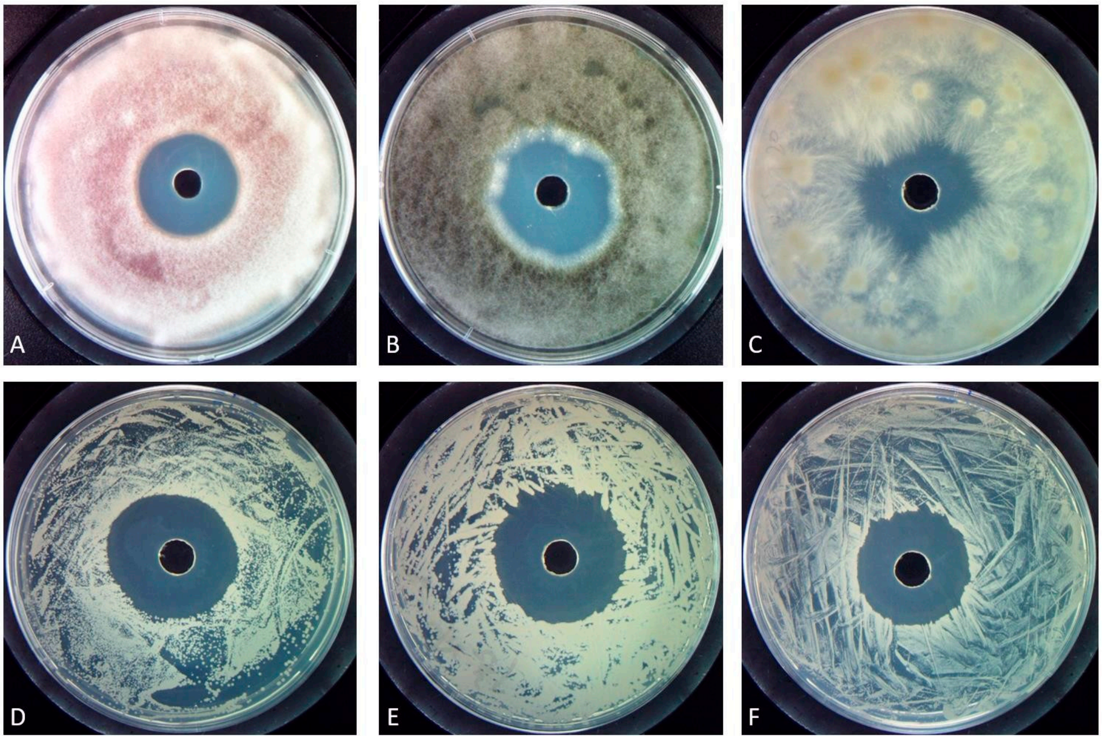

Figure S10. Antifungal activity of crude extracts from *A. spheciospongiae* against 6 pathogenic fungi.

(**A**) the conidial suspension of *Fusarium oxysporum*; (**B**) *Alternaria alternate*; (**C**) *Sclerotium rolfsi*; (**D**) the diluted broth suspension of *Candida albicans*; (**E**) *Cryptococcus neoformans*; (**F**) *Saccharomyces cerevisiae*.

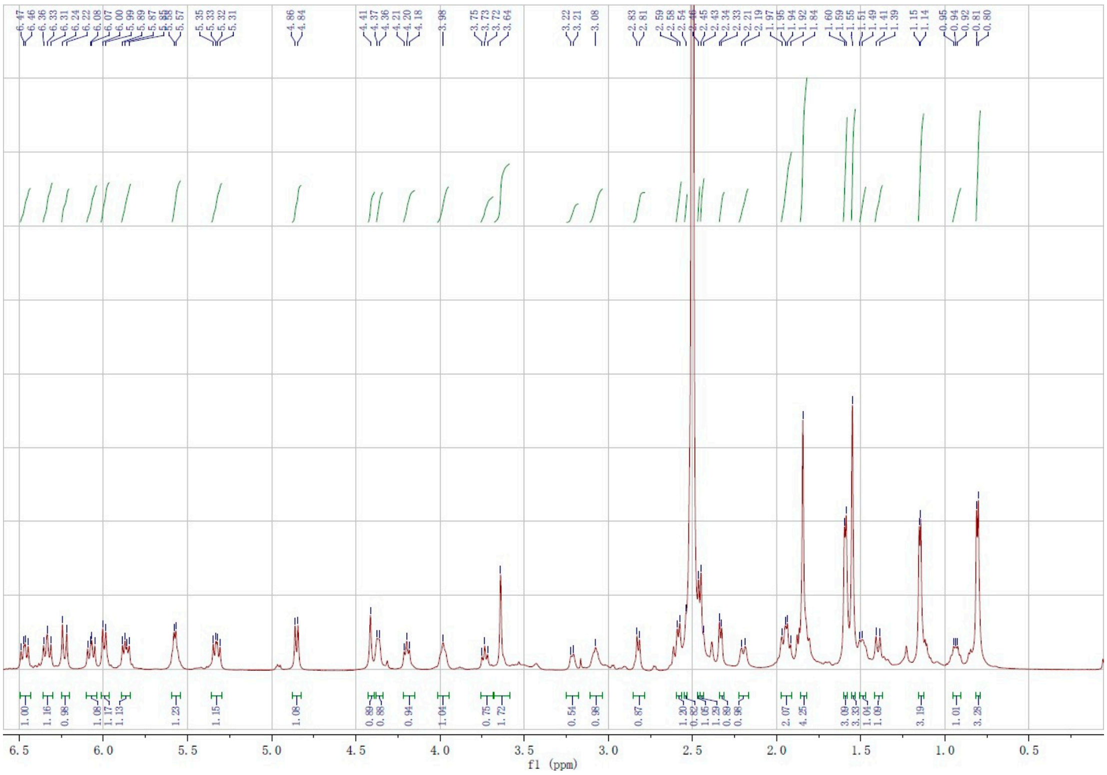

Figure S11. ^{1}H spectrum of actinospene (**1**) in DMSO-d_6.

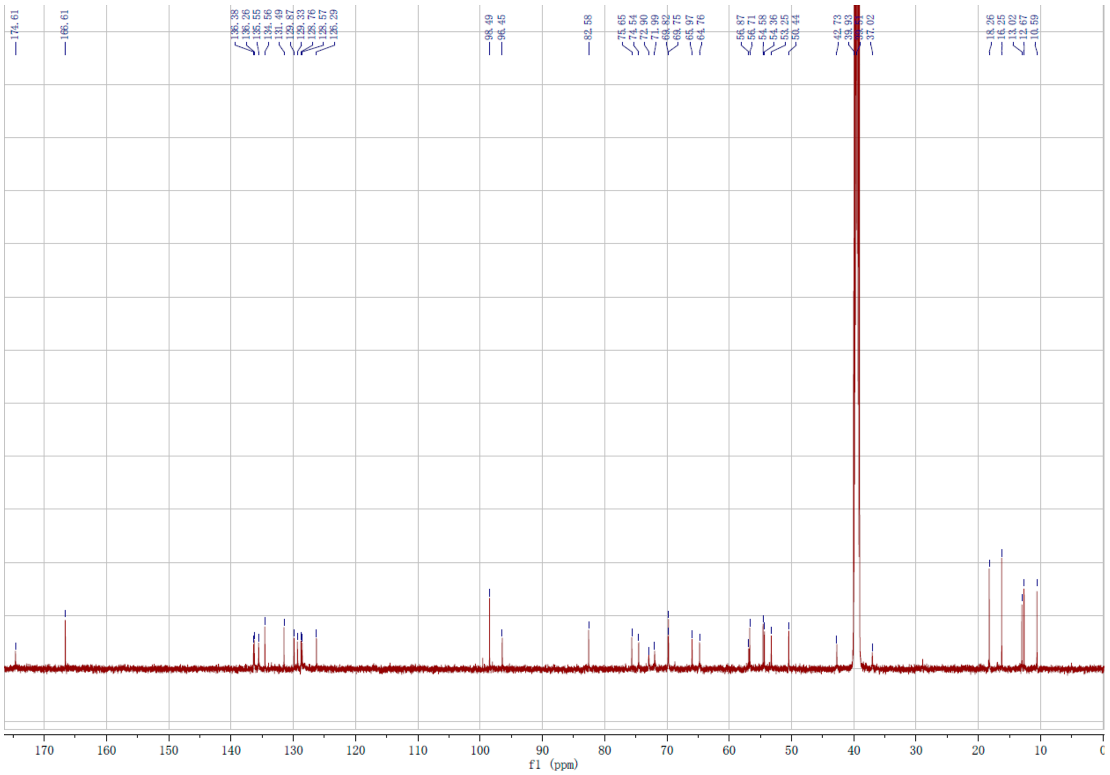

Figure S12. ^{13}C spectrum of actinospene (**1**) in DMSO-d_6.

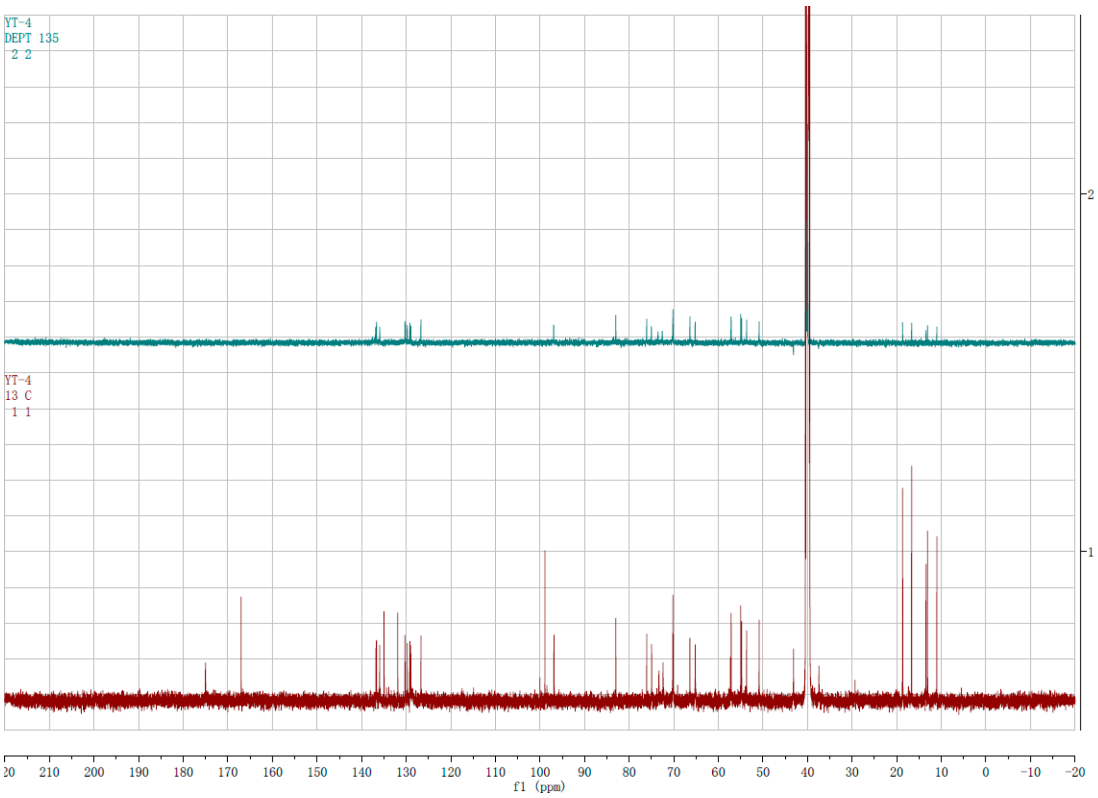

Figure S13. DEPT spectrum of actinospene (**1**) in DMSO-d_6.

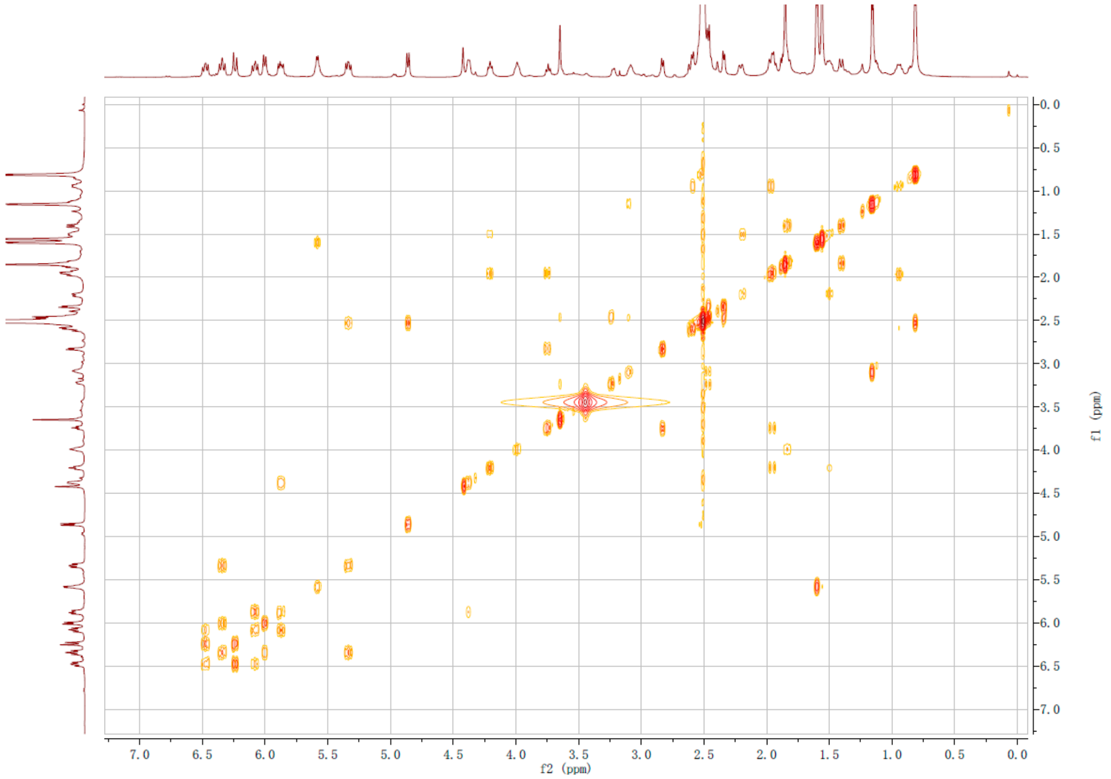

Figure S14. ^{1}H-^{1}H COSY spectrum of actinospene (**1**) in DMSO-d$_6$.

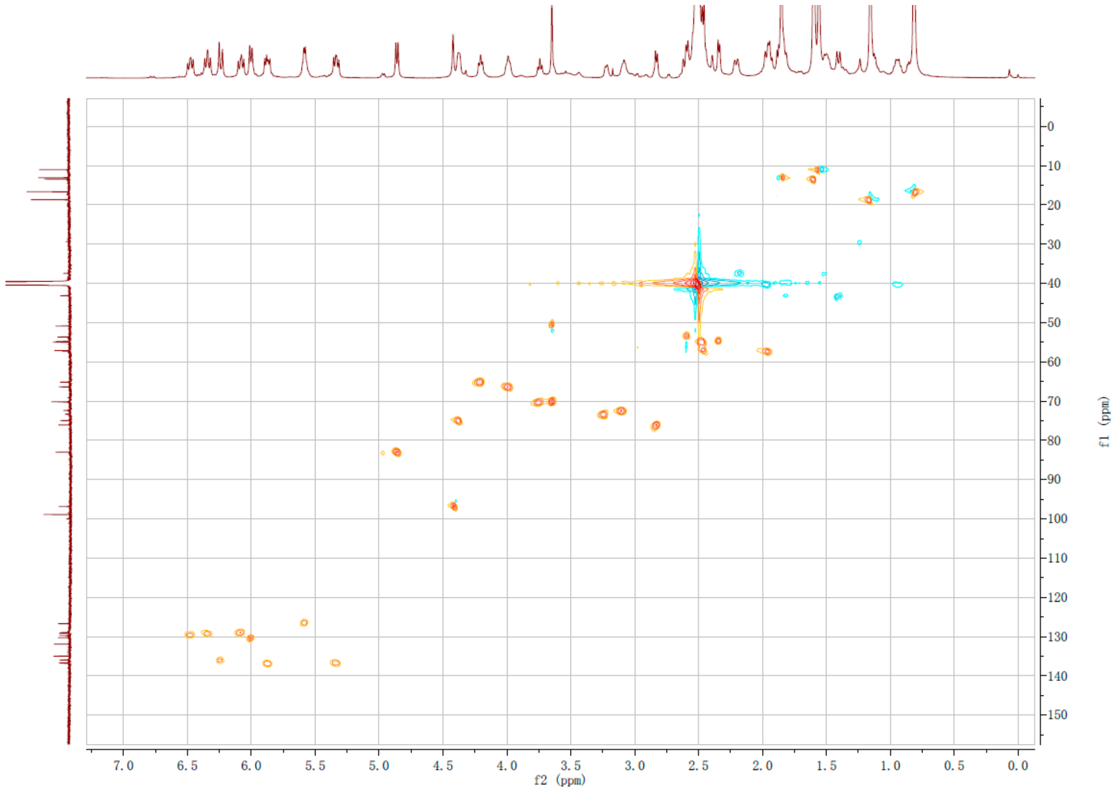

Figure S15. HSQC spectrum of actinospene (**1**) in DMSO-d$_6$.

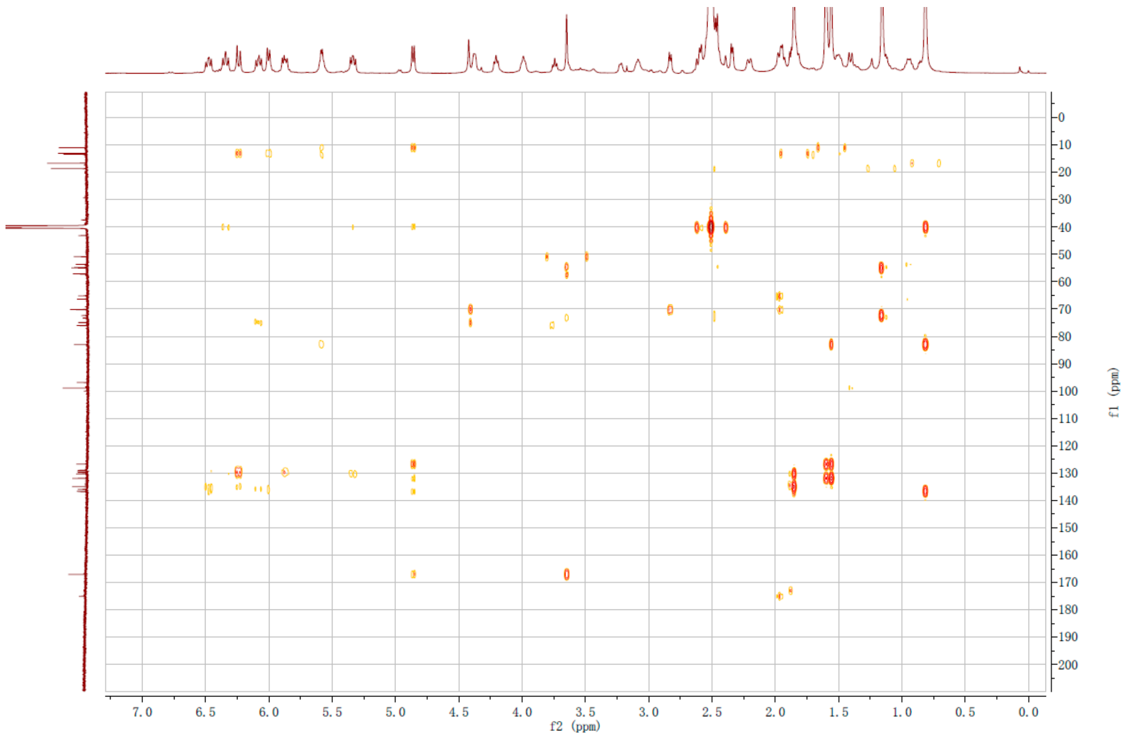

Figure S16. HMBC spectrum of actinospene (**1**) in DMSO-d_6.

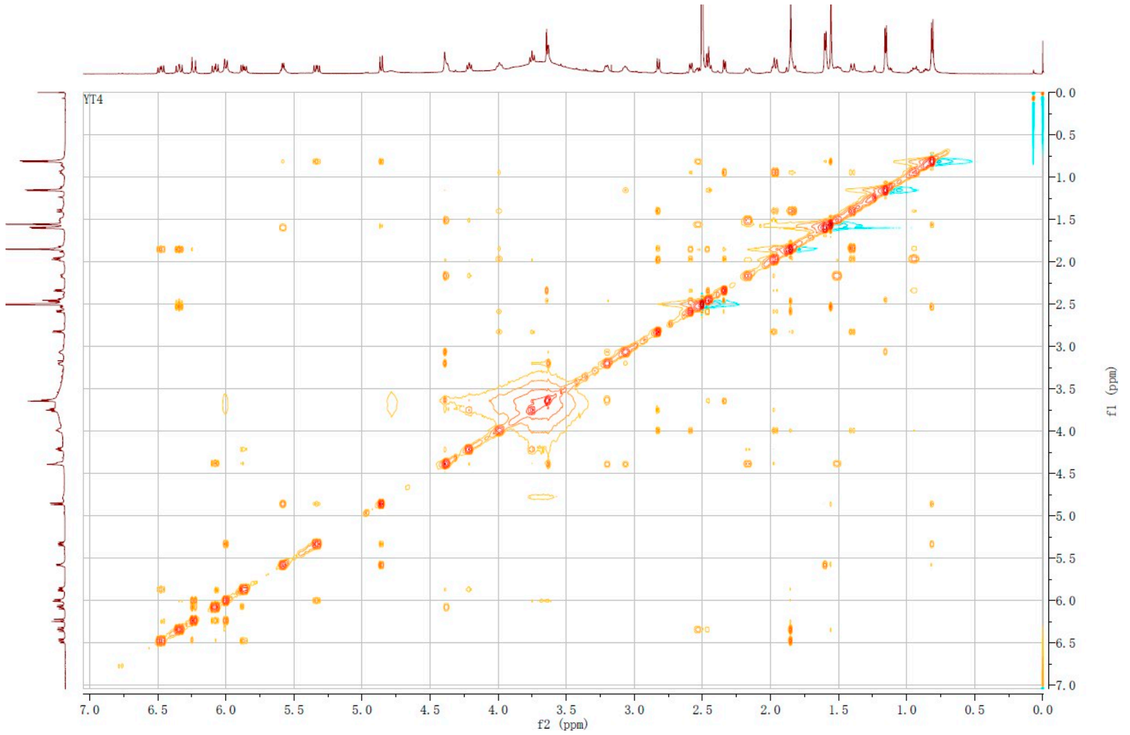

Figure S17. NOESY spectrum of actinospene (**1**) in DMSO-d_6.

References

1. Del Vecchio, F.; Petkovic, H.; Kendrew, S. G.; Low, L.; Wilkinson, B.; Lill, R.; Cortés, J.; Rudd, B. A.; Staunton, J.; Leadlay, P. F., Active-site residue, domain and module swaps in modular polyketide synthases. *Journal of industrial microbiology & biotechnology* **2003,** *30* (8), 489-494.

2. Keatinge-Clay, A., Crystal structure of the erythromycin polyketide synthase dehydratase. *Journal of molecular biology* **2008,** *384* (4), 941-953.
3. Keatinge-Clay, A. T., A tylosin ketoreductase reveals how chirality is determined in polyketides. *Chemistry & biology* **2007,** *14* (8), 898-908.
